# Helium-Based Plasma Radio Frequency Technology for Subdermal Coagulation in the Forehead: A Preliminary, Single-Center Retrospective Study

**DOI:** 10.1007/s00266-025-04737-8

**Published:** 2025-03-21

**Authors:** Aris Sterodimas, Beatriz Nicaretta, Agathi Koytsouveli, Argyris Moutafis, Grigoris Champsas

**Affiliations:** 1 Metropolitan General Hospital, 264 Mesogeion Ave, 15562 Athens, Greece; 2 Vice Head Plastic & Reconstructive Surgery Department, Metropolitan General Hospital, Athens, Greece

**Keywords:** Helium plasma and radio frequency (RF), Subdermal coagulation, Forehead lift, Brow lift, Renuvion, Skin laxity, Fibroseptal network

## Abstract

**Background:**

Helium plasma radio frequency (RF) has emerged as a minimally invasive option for treating facial laxity. This study aims to evaluate its efficacy and safety for addressing forehead and brow laxity and ptosis.

**Methods:**

In this retrospective, single-center study, medical records for 30 consecutive patients treated with helium plasma RF in the forehead and brow from March 2018 to September 2022 were reviewed. Inclusion criteria were age ≥18 years and having undergone treated in the forehead with helium plasma RF. Patients with prior or concurrent brow/forehead interventions were excluded. Primary endpoints were photographic assessments by blinded review and patient satisfaction measured via questionnaire.

**Results:**

Of the 30 patients, seven patients consented to photographic review, and all 30 participated in the patient satisfaction questionnaire. Blinded reviewers correctly identified posttreatment images in 71.4% (5/7) of cases. Patient satisfaction was high, with 100% (30/30) reporting visible improvements and willingness to recommend the procedure. Notable improvements included reduced forehead lines (56.7%), elevated eyebrows (46.7%), and a more youthful appearance (46.7%). Adverse effects were minor and expected, with bruising recorded in 20% (6/30) of patients and edema/swelling in 16.7% (5/30) of patients, all resolving within 10 days, without intervention.

**Conclusion:**

Helium plasma RF demonstrates significant potential as a minimally invasive treatment for brow and forehead laxity, with high patient satisfaction and a favorable safety profile. These preliminary findings warrant further prospective studies with larger sample sizes to confirm efficacy and long-term benefits. Helium plasma RF may provide a valuable alternative to traditional aesthetic procedures for the forehead and brow.

**Level of Evidence IV:**

This journal requires that authors assign a level of evidence to each article. For a full description of these Evidence-Based Medicine ratings, please refer to the Table of Contents or the online Instructions to Authors  www.springer.com/00266.

**Supplementary Information:**

The online version contains supplementary material available at 10.1007/s00266-025-04737-8.

## Introduction

The skin is a dynamic organ that undergoes significant changes with age, making it one of the most visible and immediately apparent indicators of aging [1]. Various interrelated cellular processes and pathways contribute to the skin’s structural integrity, with key factors including age-related changes in collagen structure and organization, decreased collagen synthesis and turnover, and increased degradation of elastin fibers. These alterations impact the skin’s tensile strength, thickness, and elasticity, leading to fine wrinkles, gradual dermal atrophy, loss of elasticity, and laxity [2, 3]. In the brow and forehead, these changes, combined with aging events across tissue layers, frequently result in patient complaints of forehead and brow ptosis and laxity in the upper eyelids.

Traditionally, these issues are addressed with excisional surgery; however, the time-consuming nature of these procedures and the risk of visible scarring have led to the development and application of less invasive endoscopic techniques for management of brow and forehead ptosis [4]. Although endoscopic lifting is less invasive than excisional surgery, it can be difficult to perform, and the results may be less durable. Nonsurgical options are also available. Brow position and forehead lines can be managed to some extent using botulinum toxin type A [5], and fillers can be used to address forehead convexity and provide support to the retro-orbicularis oculi fat. However, these modalities often do not adequately address patient concerns, especially those related to skin laxity or excess skin. Further, the forehead is associated with a high rate of filler complications [6].

Energy-based devices have also been used to address skin laxity in the upper face, including microfocused ultrasound with visualization (MFU-V) [7], laser, and radio frequency (RF) techniques. However, results with these devices can be inconsistent, and treatment is often uncomfortable for patients. Lasers and RF devices rely on bulk heating [8–15], where energy is delivered to the dermis until the desired temperature (65 °C) is reached. During this heating process, the entire volume of tissue in the treatment area is heated. This approach to thermal coagulation is somewhat indirect, often causing nontarget tissues to reach higher temperatures before adequate energy is delivered to the target tissue layer. Furthermore, the amount of time needed for collagen to coagulate is related to temperature. At 65 °C, a minimum of 120 seconds is needed for tissue contraction [16]. However, given the established benefits of thermal coagulation and the predictable nature of tissue contraction and skin tightening [17, 18], there is significant interest in identifying effective modes of coagulation that may serve as a better alternative to excisional procedures.

Recently, helium-based plasma RF (Renuvion, Apyx Medical, Clearwater, FL, USA) has emerged as a technology capable of directly deliver thermal energy to the subcutaneous fascia and septal connective tissues. The device is cleared by the United States Food and Drug Administration for the subcutaneous delivery of RF energy and/or helium plasma where coagulation/contraction of soft tissue is needed, and more specifically for use in the neck and submental area to improve the appearance of lax skin and in the body for aesthetic body contouring [19]. The mechanism of the device is distinct as it does not rely on bulk heating. Instead, it locally heats tissues through two mechanisms as the electrode at the end of the device’s handpiece is passed through the subcutaneous layer.

First, when helium gas is passed over an electrode that has been energized by RF, helium plasma is generated. The production of the plasma beam generates heat through ionization and neutralization of helium ions. Second, a portion of the RF energy used to energize the electrode generates heat as it passes through the plasma beam to surrounding tissues, heating the tissues via resistance. The energy delivery is localized to the area around the electrode, and as the device electrode moves through the tissue plane, the plasma beam quickly heats different tissues near the tip of the device, instantly heating tissues to temperatures >85 °C. At this temperature, complete protein coagulation occurs in just 0.04 sec [20]. Because this type of heating requires much less time and is restricted to the area around the handpiece tip, adjacent tissues are not heated, and the treated area cools rapidly (external temperature increases are ≤3.6 °C [20]), allowing multiple passes in a relatively short treatment time.

In the retrospective study presented here, medical records were reviewed for patients who were treated with minimally invasive plasma RF in the forehead and brow to address laxity and aging in the upper face. The results highlight the potential for helium plasma RF in managing forehead and brow ptosis and upper lid laxity.

## Methods

### Study Design

In this retrospective, single-center study, the medical records of patients who received a single treatment with the helium plasma RF system for subdermal coagulation in forehead between March 2018 and September 2022 were evaluated. Inclusion criteria included age over 18 years and treatment with helium-based plasma RF for subdermal coagulation in the forehead and to elevate the brow. Patients who had undergone other surgical or nonsurgical interventions (e.g., other energy-based treatments or injectables) in the upper face within 6 months prior to treatment or during the follow-up period were excluded. Patients meeting the study criteria were asked if they would consent to having their photographs reviewed and/or completing a patient satisfaction questionnaire (PSQ). This study was approved by the Ethics Committee for the Metropolitan General Hospital, Athens, Greece, and all patients shown here consented to publication of their photographs. All procedures were conducted in accordance with the ethical standards of the ethics committee and with the 1964 Helsinki Declaration and its later amendments or comparable ethical standards.

### Efficacy and Safety Endpoints

The primary endpoint in this study was improvement in the brow and forehead, as determined by a masked, qualitative assessment of photographs taken 90+ days after treatment compared to baseline. Three blinded reviewers assessed randomized before-and-after photographs and were asked to identify the “after” photograph. Reviewers were informed of the treated area and instructed to specifically consider changes in lines and/or wrinkles, laxity, and overall appearance of the treatment area. Successful identification of the “after” image was defined as correct posttreatment image selection by at least two of three reviewers.

The patient satisfaction questionnaire (PSQ) was completed by study patients either over the phone or via email. The questions focused on patient perceptions of aesthetic changes, satisfaction, and willingness to recommend the treatment to others.

Complications and adverse events noted in patient charts were compiled during chart review.

### Treatment Methodology

**Fig. 1:**
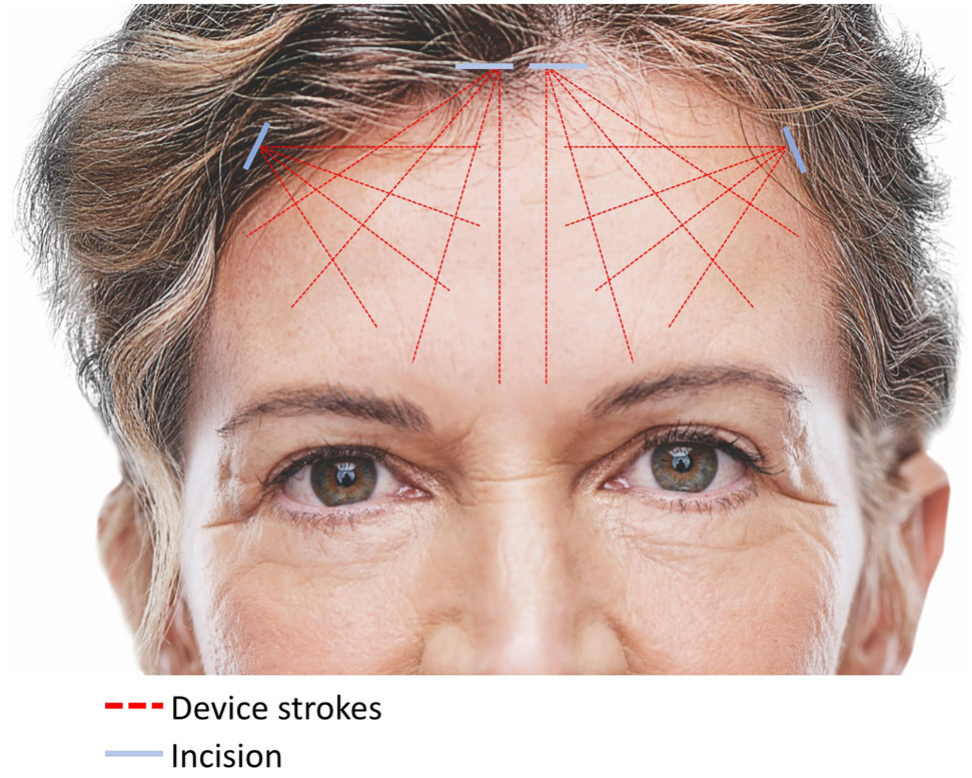
An example of helium plasma RF strokes applied during forehead treatment (dashed lines, red). Incision points are shown as solid lines (blue). Treatment strokes can be adjusted beyond those paths shown to accommodate different brow positions and whether the patient is in need of more medial or lateral lift

**Fig. 2:**
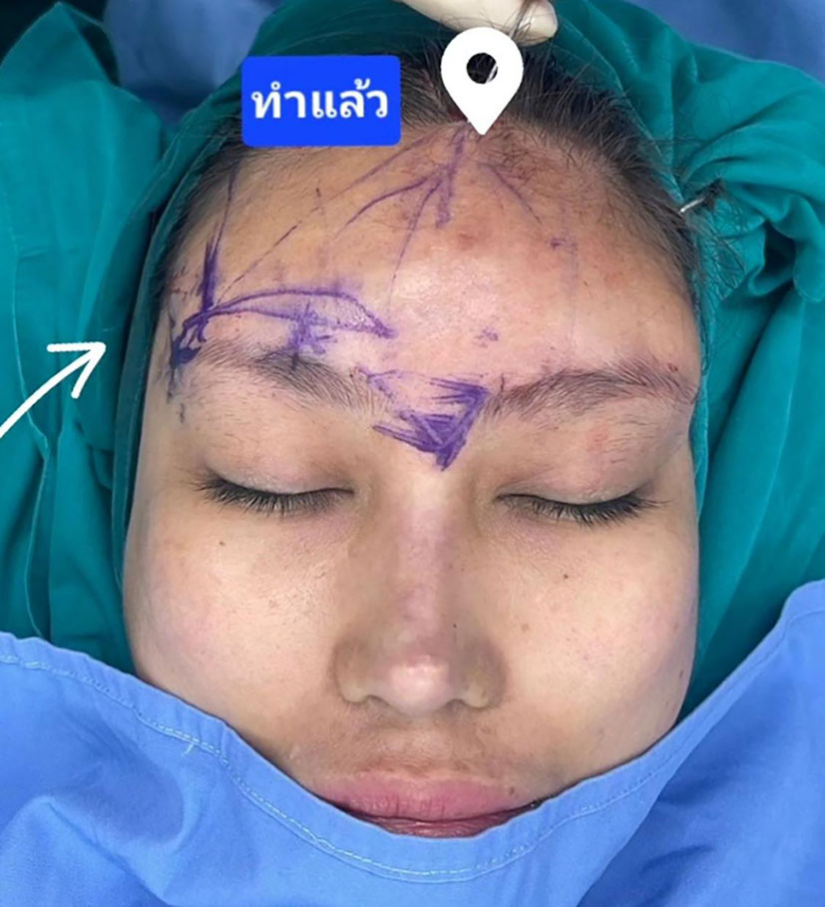
An image showing the action of helium plasma RF of the brow. The patient has been treated on the left side of the face. Note the difference in brow elevation and brow shape

After administering anesthesia, two 3–4 cm incisions were made behind the hairline: one just lateral to the medial forehead on each side (one 1 cm from the midline and the other 4 cm from the midline) (Fig. 1). The treatment area was then infused with 30 cc of tumescent solution (0.2% lidocaine and 5 µg/mL epinephrine in normal saline) per side to ensure sufficient infiltration, an aspect of treatment which is important for optimal outcomes. After several minutes, during which the anesthetic was permitted to affect the treatment area, the area was undermined with a 1.9-mm diameter cannula. The helium plasma RF device was then applied to forehead and above the brow. The area was treated with between three and five passes at each incision point, based on patient skin quality and anatomy, within the supraperiosteal or subgaleal plane. Patients with thinner skin were treated with fewer passes to minimize the chances of overheating the epidermis. An example of the individual strokes comprising each treatment pass is shown in Fig. 1; however, strokes can be modified based on the areas of the eyebrows that need to be lifted. Within the patient group described here, several of the patients received treatment in the area above the lateral brow as well. The degree of lift achieved can be appreciated in Fig. 2 where an intraoperative photograph of a patient is shown where half of the brow has been elevated. Settings were 65% power, 1 L/min of helium flow, and activation speed of 1–3 cm. Importantly, strokes were between 1 cm and 3 cm apart to maintain both efficacy and safety. Following treatment, all excess helium gas was manually expressed from the treated tissue and aspirated. A video of the procedure is shown in Video 1. It is important to avoid the surpraorbital and supratroclear nerves; an assistant can protect the area during the procedure. By treating in the supraperiosteal plane, the frontal branch of the facial nerve is avoided.

Following the procedure, the position of the lateral and medial brow was fixed using tape, and patients were instructed to leave the tape in place for one week. Twenty-four hours following the procedure, patients were permitted to wash their face and to apply makeup. As per standard practice for forehead/brow procedures, patients were seen on days 1, 7, and 40 post-procedure. Follow-up photographs used in the photographic evaluation were taken at the time patients were contacted and consented to participation in this retrospective study.

## Results

Of the medical records reviewed for patients who had been treated with helium plasma RF, a total of 30 patients met the criteria for participation in the study. Study demographics and details of the surgical variables and technique for these 30 patents are presented in Table 1. For all patients in the analysis, treatment was limited to the forehead, above the brow; with none of the patients receiving treatment in the periorbital area. Twenty-four of 30 patients received local sedation, and six underwent general anesthesia. The median helium plasma RF settings for the forehead/brow treatments were 65% power (range: 60–75), 1 L/min helium flow rate, four retrograde passes (range: 3–5) at each incision point, and 2.4 kJ of energy applied (range: 1.6–2.8).

**Table 1:** Demographics and treatment characteristics

Characteristic	Enrolled patients (*N*=30)
*Sex, n (%)*	
Male	25 (83)
Female	5 (17)
*Age (years)*	
Mean ± SD (min, max) Median	54 ± 10 (27, 69) 55
*Renuvion power, n (%)*	
60	1 (3.3)
65	22 (73.3)
70	6 (20.0)
75	1 (3.3)
*Number of passes*	
3	8 (26.7)
4	17 (56.7)
5	5 (16.7)
*Energy (kJ)*	
Mean ± SD (min, max) Median	2.2 ± 0.4 (1.6, 2.8) 2.4

**Fig. 3:**
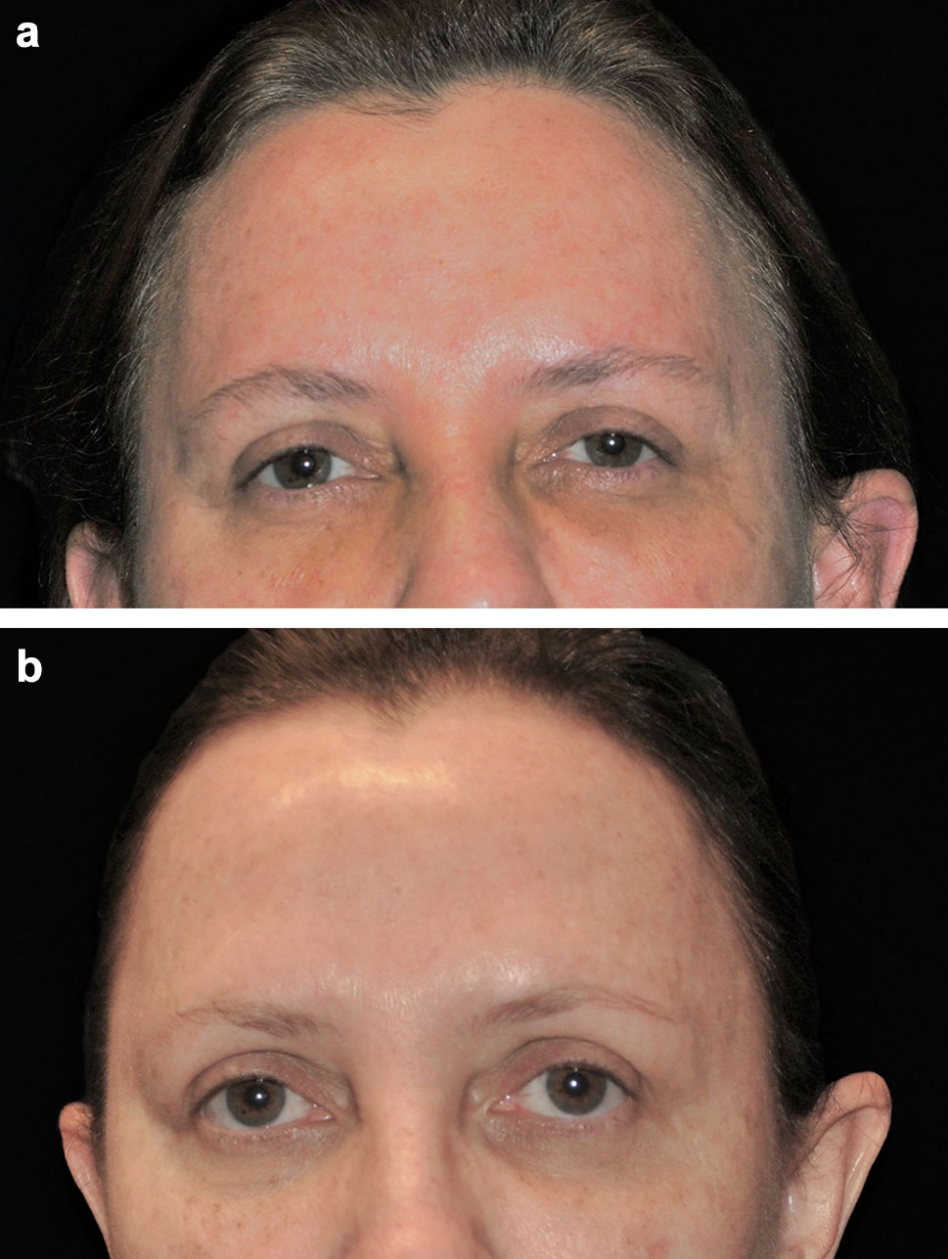
A 56-year-old female patient before (**a**) and 32 months after treatment of the brow and forehead (**b**). Treatment settings: 65% power, 1 L/min, 2 kJ, three passes. Note the natural-looking-results and degree of lift achieved

**Fig. 4:**
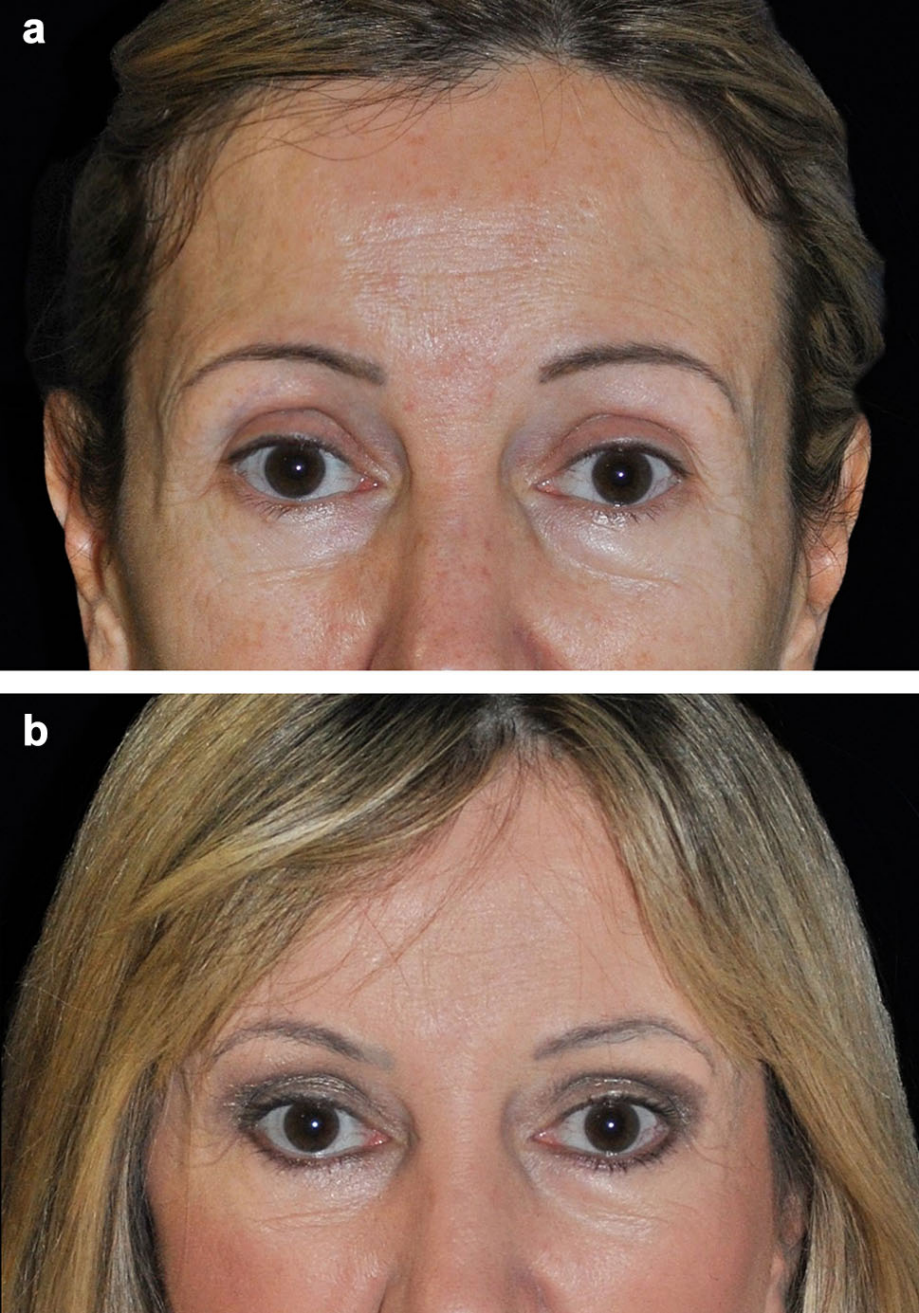
A 65-year-old female patient before (**a**) and 33 months after treatment of the brow and forehead (**b**). Treatment settings: 65% power, 1 L/min, 2.7 kJ, four passes. Note the improved eyebrow shape and lift in the medial brow

**Fig. 5:**
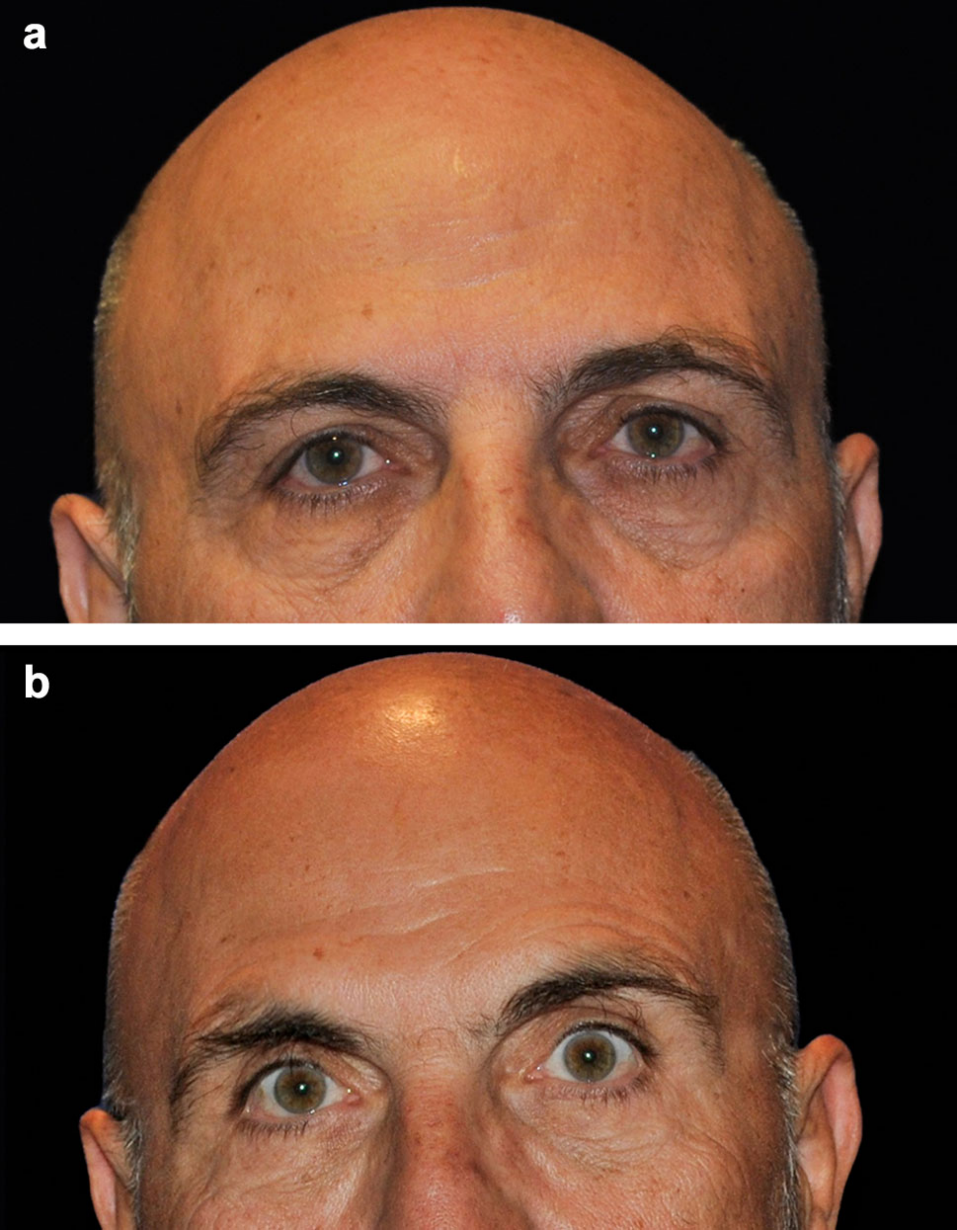
A 68-year-old male patient before (**a**) and 46 months after treatment (**b**) of the brow and forehead. Treatment settings: 65% power, 1 L/min, 1.8 kJ, three passes. Note the lifting in the lateral eyebrow

Out of the 30 patients treated, seven of these patients consented to the use of their photographs. These photographs were taken an average of 27 months following the procedure (range: 3–46 months). Independent photographic reviewers correctly identified the “after” photographs in 71% (5/7) of cases. Examples are shown in Figs. 3, 4, and 5.

All 30 subjects consented to participate in the PSQ, with a mean follow-up time of 24 months (range: 1–57). All patients (100%, 30/30) reported seeing an improvement in the area treated with the helium plasma RF device and stated they would recommend the procedure to a friend (Table 2). When they were asked to select from a list of noticed improvements, the greatest improvement reported was the reduction of visible forehead lines (56.7%, 17/30), followed by eyebrow elevation (46.7%, 14/30) and feeling that they looked younger (46.7%, 14/30). All patients (100%) reported satisfaction with the treatment, with a majority (63.3%, 19/30;) reporting they were “very satisfied.”

**Table 2:** Patient satisfaction questionnaire results

Question/characteristic	Result, *n* (%) (*N*=30)
*Improvement in the area that was treated with Renuvion?*	
Yes	30 (100)
My forehead lines seem improved	17 (56.7)
I feel like I look younger	14 (46.7)
My eyebrows seem elevated	14 (46.7)
Improvement in wrinkles	13 (43.3)
Less sagging skin	12 (40.0)
Smoother skin texture	11 (36.7)
My upper face looks better	11 (36.7)
I feel like I look more refreshed	9 (30.0)
My upper eyelid feels less heavy	8 (26.7)
My eyes seem more open	7 (23.3)
My crow’s feet seem improved	6 (20.0)
More even skin tone (color)	5 (16.7)
My skin on lower eyelid seems tighter	3 (10.0)
No	0 (0)
*Characterize satisfaction with the treatment*	
Very satisfied	19 (63.3)
Satisfied	9 (30)
Slightly satisfied	2 (6.7)
Neither satisfied or dissatisfied	0 (0)
Dissatisfied	0 (0)
Very dissatisfied	0 (0)
*Would you recommend procedure to friends and family?*	
Yes	30 (100)
No	0 (0)

**Fig. 6:**
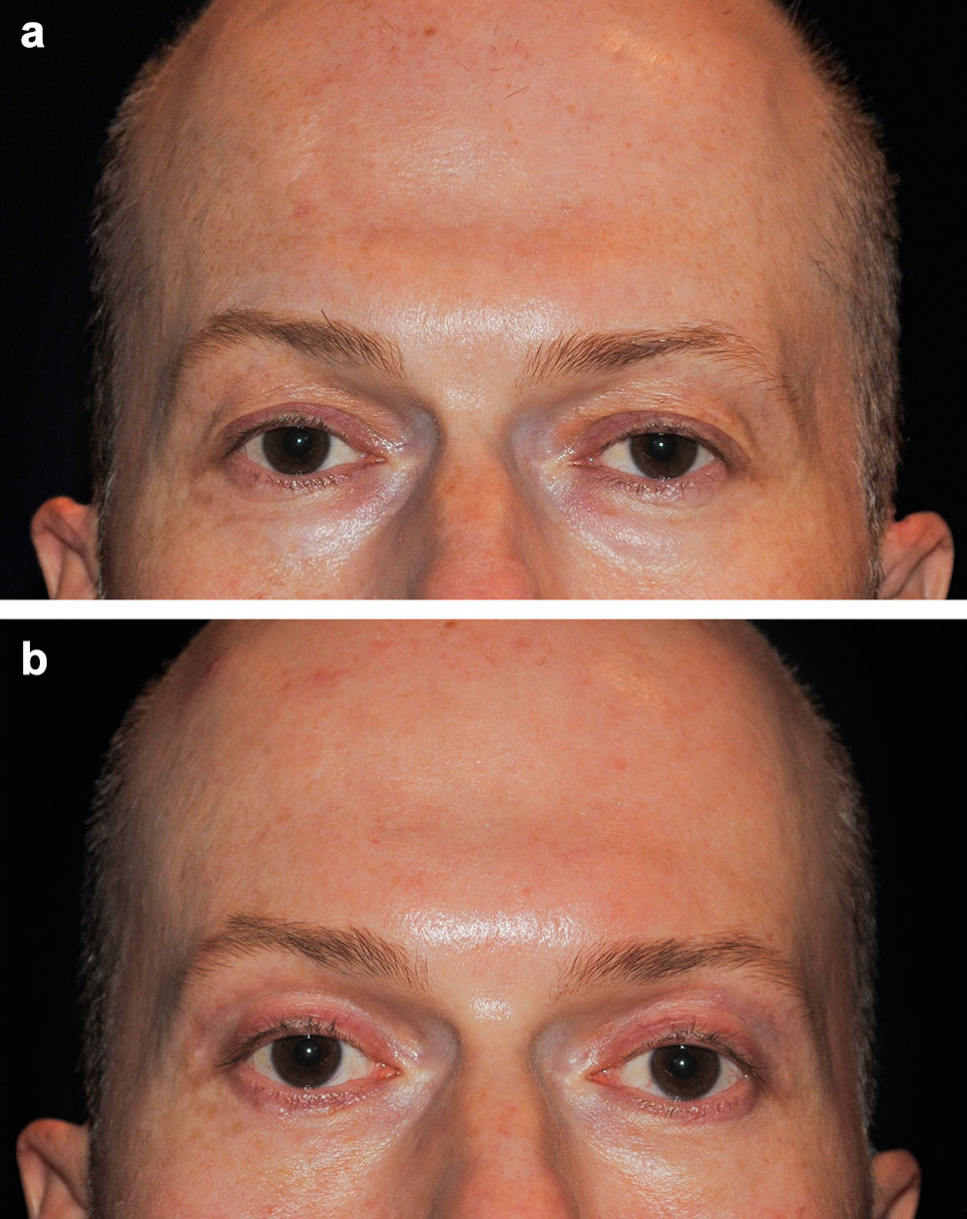
A 46-year-old male patient before (**a**) and 5 months after treatment (**b**) of the brow and forehead. Treatment settings: 65% power, 1 L/min, 1.8 kJ, three passes. Note the lifting in the medial and lateral eyebrow

In this study, seven patients (23.3%, 7/30) experienced a total of 11 expected treatment effects (Table 3). These events were limited to bruising (20%, 6/30 patients) and edema/swelling (16.7%, 5/30 patients), both of which are common and anticipated outcomes in procedures involving tissue undermining. All events were resolved or nearly resolved by the 10-day follow-up visit without intervention, with 2 events resolving by day 3, 1 event by day 5, 4 events by day 8, and 3 events by day 10. No serious adverse events were reported (Fig. 6).

**Table 3:** Adverse events observed and duration until resolution

		Duration (days)
Adverse event	Total events	Average (min, max)
Bruising	6	7.0 (3, 10)
Edema	5	7.2 (5, 10)
Total	11	7.1 (3, 10)

## Discussion

Helium plasma RF is a unique device in the treatment of armamentarium for addressing laxity in the brow and forehead. It is minimally invasive and directly affects the fascia and septal network to contract collagen. The technology is efficient, with treatments taking approximately 10–15 minutes per side, and in this study, 83% of patients [25/30] underwent other surgical procedures at the same time. Unlike other methods that rely on bulk heating, helium plasma RF rapidly denatures proteins and coagulates tissues as the handpiece is passed through the target tissue plane. The target tissue instantly reaches 85 °C, achieving rapid tissue contraction with minimal risk of overheating the overlying skin [20]. These characteristics, along with the preliminary evidence presented here, highlight the potential of the device to serve as a reliable alternative to bulk heating technologies, excisional or endoscopic technologies, offering a potentially more predictable method for achieving tissue contraction in the upper face. Additionally, the fine control provided by helium plasma RF is advantageous in addressing patient brow shape. For example, in Fig. 4, the improved medial and lateral position of the brow can be appreciated. It should be noted that the treatment strokes can be adjusted beyond those paths shown in Fig. 1 to accommodate different brow positions and whether the patient is in need of more medial or lateral lift.

In terms of efficacy, the small number of patients who consented to photographic review (7/30) resulted in a limited sample size. However, the 71% (5/7) rate of correct identification of “after” photographs by independent reviewers aligns within the range of expectations seen in other studies for minimally invasive aesthetic medical devices [21, 22]. The data also reflect a high level of patient satisfaction (100%; 30/30) among the larger group of patients. Despite the small sample size, the study provides valuable insights into the device settings used for treating the forehead and brow, as well as the associated safety profile. These findings pave the way for future research in this area. Notably, 100% of patients reported improvement in the treated area (Table 2). Specifically, patients noted improvements in forehead lines (56.7%, 17/30), wrinkles (43.3%, 13/30), elevated eyebrows (46.7%, 14/30), a more youthful appearance (46.7%, 14/30), and reduced sagging skin (40%, 12/30).

With this supportive evidence, helium plasma RF may fulfill a significant unmet need in brow lifting. Patients almost universally prefer minimally invasive techniques whenever possible. However, relaxing the depressor muscles in the brow with botulinum toxin is not only temporary, but also does not address overlying skin tone. Therefore, the lift achievable with botulinum toxins often becomes insufficient over time. Surgical techniques, which have evolved significantly, range from open coronal brow lifts to limited-incision endoscopic techniques. While recent endoscopic techniques offer a safer, less invasive approach, helium plasma RF provides a minimally invasive option that does not require excision and fixation, thereby reducing the risk of alopecia or scarring. Further, the technique is more technically straightforward and does not require the use of a scope. The rapid recovery time, minimal downtime, and reduced risk of complications associated with helium plasma RF further enhance its appeal. In the future studies, it will be important to include comparators, as the durability of helium plasma RF compared to other modalities will be an important aspect of treatment. The current study includes a range of follow-up out to 47 months. While the outcomes here appear to be durable, the small number of patients in the study and the lack of a comparator prevent a direct comparison to other devices/procedures. Taken together, the results indicate that helium plasma RF has the potential to serve an important role for both patients and practitioners in the field of aesthetic medicine.

Importantly, the only reported adverse events were expected treatment effects, with bruising and edema occurring in 23.3% (7/30) of patients (Table 3). Although the severity was not specifically rated, most instances were relatively mild and resolved within 10 days; no serious adverse events were reported. These data suggest that the safety profile of helium plasma RF makes it an excellent option for addressing skin laxity in the brow/forehead.

Given the retrospective design and small sample size of this study, both of which are limitations, future research will be necessary to better assess the efficacy of helium plasma RF in the forehead and brow across a broader range of patients. Because skin response to RF energy may vary with factors like age, Fitzpatrick skin type, and ethnicity, more diverse sample would make the findings more generalizable and better inform treatment protocols across different patient populations. In addition, a larger study with an analysis of factors influencing patient satisfaction (e.g., AEs, patient features) would be informative for clinical practice. Future studies should include diligent photography to ensure the results can be accurately appreciated and measured. While supraperiosital application of helium plasma RF energy should permit the frontal branch of the facial nerve to be avoided, a much larger sample set will be needed in order to more clearly define safety and risk of injury to the nerves and vasculature of the forehead. Additionally, the scope of the evaluation could be expanded to include fine lines, wrinkles, and overall skin quality.

## Conclusion

This retrospective study highlights the potential of helium plasma RF technology as a minimally invasive procedure for addressing laxity and aging in the forehead and brow. The effectiveness of helium plasma RF in reducing visible forehead line, elevating eyebrows, and providing a more youthful appearance suggests it could be a valuable alternative to traditional surgical and nonsurgical procedures for addressing laxity and ptosis in the brow, forehead, and upper eyelids. Further prospective studies with larger sample sizes and rigorous photographic documentation are warranted to confirm these findings.

## Supplementary Information

Below is the link to the electronic supplementary material.

Supplementary file1 (MP4 122770 KB)
